# Effect of oral hypotonic water and isotonic saline on physiological intestinal ^18^F‐FDG uptake in PET/CT imaging

**DOI:** 10.1002/acm2.70431

**Published:** 2025-12-29

**Authors:** Shijia Weng, Yuan Li, Xiaoren Liu, Qian Wang

**Affiliations:** ^1^ Department of Nuclear Medicine Peking University People's Hospital Beijing China

**Keywords:** 18F‐FDG PET/CT, intestine, osmotic pressure, physiological uptake

## Abstract

**Purpose:**

Hydration of patients before the PET/CT examination may lead to non‐specific FDG accumulation in the intestine. This study aims to explore the influence of isotonic saline and hypotonic water on physiologic intestinal ^18^F‐FDG uptake in PET/CT imaging.

**Methods:**

185 patients were included, 82 patients were randomized to receive oral administration of isotonic saline and 103 patients received oral administration of hypotonic water before examination. About 32 patients in the isotonic group and 19 patients in hypotonic group underwent repeated PET/CT with pre‐administration of hypotonic water, and the intra‐individual comparative analysis was performed for the first and second examinations. Segmental uptake patterns were documented and volumetric regions of interest were delineated. The SUVmax of intestinal segment and the SUVmean of liver were recorded.

**Results:**

No significant differences existed in baseline characteristics between the two groups. The isotonic group demonstrated lower physiological uptake incidence and target‐to‐background ratio (TBR) in all segments except jejunum and ascending colon (*p *< 0.05). Physiological uptake incidence in jejunum showed no intergroup difference, whereas the TBR of isotonic group showed lower tendency. Notably, the isotonic group exhibited higher physiological uptake incidence (*p *< 0.001) in the ascending colon, though no significant difference existed in TBR between two groups. These results were similar in intra‐individual comparative analysis of the first and second examinations. Moreover, constipation was positively associated with FDG uptake in the ileum, diarrhea was positively associated with FDG uptake in the transverse colon, ascending colon and rectum. Bone tumors and gynecological tumors were positively correlated with FDG uptake in the ascending colon and transverse colon. BMI was negatively correlated with FDG uptake in the duodenum, ileum, jejunum, and sigmoid colon.

**Conclusion:**

Pre‐administration of isotonic saline resulted in lower physiologic ^18^F‐FDG uptake in different intestine segments (except ascending colon), which is helpful for accurate assessment of gastrointestinal diseases.

## 1 INTRODUCTION

The intestine is the longest duct in the digestive system with a total length of approximately 7 m, which comprises the duodenum, jejunum, ileum, cecum, colon, and rectum.^1^, ^2^ The mucosal layer of the intestine is covered with intestinal villi, which primarily facilitates the digestion and absorption of food. Chyme is further digested into glucose, amino acids, and other nutrients. Meanwhile, the remaining waste forms feces, which are stored in the left colon.^3^, ^4^ As one of the most common malignant tumors, colorectal cancer was reported as the third most common cause of cancer in both men and women worldwide, with a high mortality rate.^5^, ^6^ However, early‐stage colorectal cancer often lacks specific symptoms, making early diagnosis challenging.

Whole body positron emission tomography/computed tomography (PET/CT) imaging is an integrated diagnostic modality combining PET and CT. PET/CT imaging enables visualization of both anatomical structural changes and metabolic activity in different tissues, which offers significant advantages in the early screening, diagnosis, staging, detection of recurrence or metastasis, and treatment evaluation of intestinal tumors.^7^, ^8^ 2‐deoxy‐2‐[^18^F]fluoro‐d‐glucose (^18^F‐FDG) is the most commonly used tracer, which exhibits physiological distribution within the intestine as a glucose analog.^9^, ^10^ However, due to the large surface area and unstable filling state of intestine, physiological glucose uptake may affect systemic metabolic assessments, potentially masking pathological uptake caused by intestinal tumors, inflammation, or other conditions.^11^, ^12^

Studies have reported that pre‐examination factors such as diet, metformin use, and mannitol administration could influence physiological intestinal ^18^F‐FDG uptake.^13^, ^14^, ^15^ Additionally, osmotic pressure was reported to play a critical role in intestinal glucose metabolism.^16^, ^17^ However, the impact of pre‐examination osmotic pressure on intestinal ^18^F‐FDG uptake has not been well established. In this study, we compared the physiologic ^18^F‐FDG uptake in different segments of intestine in patients with two different osmotic pressure.

## 2 MATERIALS AND METHODS

### 2.1 Subjects

This study enrolled 241 patients at our PET/CT center from March to April 2023, 56 patients were excluded for gastrointestinal disease, history of surgery, metformin use within 7 days before examination or abnormal fasting glucose level (exceeded 11.1 mmol/L) before examination,^18^, ^19^, ^20^ and ultimately 185 patients were enrolled and completed at least one year of follow‐up with no documented gastrointestinal complications. All patients were uniformly treated with adequate prehydration 60 min prior to FDG injection, 82 patients were randomly assigned to oral administration of 500 mL commercially available 0.9% isotonic saline (isotonic group), while the remaining 103 patients received oral administration of 500 mL commercially available purified water (hypotonic group). During the follow‐up period, 51 patients (32 from the isotonic group and 19 from the hypotonic group) underwent repeated FDG PET/CT examinations for evaluation of medical treatment. They all received 500 mL water before examination. This study was reviewed and approved by our Institutional Ethics Committee, and all methods were adhered to the ethical principles of the Declaration of Helsinki. All participants signed written informed consent.

### 2.2 PET/CT data acquisition

All patients maintained a fasting period (≥6 h) prior to examination, with blood glucose levels strictly controlled below 11.1 mmol/L. All patients underwent PET/CT examination 50∼70 min after intravenous administration of ^18^F‐FDG (Beijing Atom HighTech Co., Ltd.) with a standardized dose of 4.81∼5.55 MBq per kg. PET/CT studies were performed from the head to the mid‐thigh (with extended scanning to the feet when clinically needed) with an image duration of 2.5 min per bed position for the emission scans. A general electric (GE) discovery VCT PET/CT system operated in a three‐dimensional mode was used. A low‐dose attenuation CT (140 kV) was used for attenuation correction of the PET data and for image fusion.

### 2.3 PET/CT data analysis

Visual analysis of intestinal ^18^F‐FDG uptake was independently performed by three experienced nuclear medicine physicians (more than 10 years of experience) through blinded consensus reading sessions. PET/CT images were systematically evaluated for uptake localization (specific intestinal segments) and morphological characteristics. Triplanar reconstructions were integrated with simultaneous review of PET, CT, and fused PET/CT datasets, which enabled precise tomographic mapping of intestinal tracer distribution. Positive physiological intestinal uptake was defined as a higher intestinal FDG uptake than the peripheral background on PET images.

One another experienced nuclear medicine physician performed quantitative analysis based on volumes of interest (VOI), which included duodenum, proximal jejunum, distal ileum, ascending colon, transverse colon, descending colon, sigmoid colon, and rectum. VOI encompassed the intestinal wall and luminal contents, with careful exclusion of extraluminal structures. Maximum standardized uptake value (SUVmax) for each intestinal segment was also recorded. Moreover, target‐to‐background ratio (TBR) was calculated as: (Intestinal segment SUVmax)/(Hepatic SUVmean).

PET/CT imaging features of isotonic group and hypotonic group were compared, which included uptake intensity and uptake patterns. For those undergoing repeat scans (32 patients in isotonic group and 19 patients in hypotonic group), the intra‐individual differences of PET/CT imaging between first and second scans were compared. For the intestinal segments with differences in uptake, further analysis was performed to find potential factors influencing FDG uptake, including the patient's general clinical information, disease type, laboratory tests, and interval time between agent injection and imaging, etc.

### 2.4 Statistical analysis

SPSS 27.0 software was used for data analysis. An independent *t*‐test was used to analyze the TBR differences between intestinal segments in the isotonic group and hypotonic group. A paired *t*‐test was used to analyze the TBR differences of intestinal segments between first and second examinations in isotonic and hypotonic groups. The multiple linear regression analysis was used to analyze the influencing factors in intestinal segments with differences in [18F]‐FDG uptake, and regression coefficient are presented. When *p *< 0.05, it is considered statistically significant.

## 3 RESULTS

### 3.1 Patient characteristics

Patient demographics are listed in Table 1. There are no significant differences in the gender and history of diabetes between two groups. The mean age, height, weight, body mass index (BMI), blood glucose on the day of examination, and the time from injection to collection are not statistically different between two groups (*p *> 0.05). The isotonic group included 82 cases (lung cancer 26, hematological diseases 21, connective tissue diseases 11, bone tumors 7, reproductive and urinary system tumors 7, infectious diseases 5, and other malignant tumors 5). The hypotonic group included 103 cases (lung cancer 41, hematological diseases 21, connective tissue diseases 5, infectious diseases 11, bone tumors 9, reproductive and urinary system tumors 9, other malignant tumors 7).

**TABLE 1:** Demographic information of patients included in this study.

	*N*	Age	% Female	% with Diabetes	Glucose (mmol/L)	Height (cm)	Weight (kg)	BMI (kg/m^2^)	The FDG circulation time (min)
Isotonic group	82	57.8 ± 17.5	50	2.3	5.8 ± 0.9	166.8 ± 8.4	66.1 ± 12.6	23.7 ± 3.7	58.4 ± 11.5
Hypotonic group	103	55.2 ± 17.6	48	5.8	5.5 ± 0.9	166.8 ± 8.2	66.6 ± 14.2	23.8 ± 4.0	61.3 ± 15.5
*p*‐value	–	0.326	0.773	0.291	0.281	0.969	0.785	0.811	0.138

Values are presented as mean ± standard deviation (SD).

### 3.2 Comparative analysis of FDG uptake in intestine segments between isotonic and hypotonic groups

**FIGURE 1:**
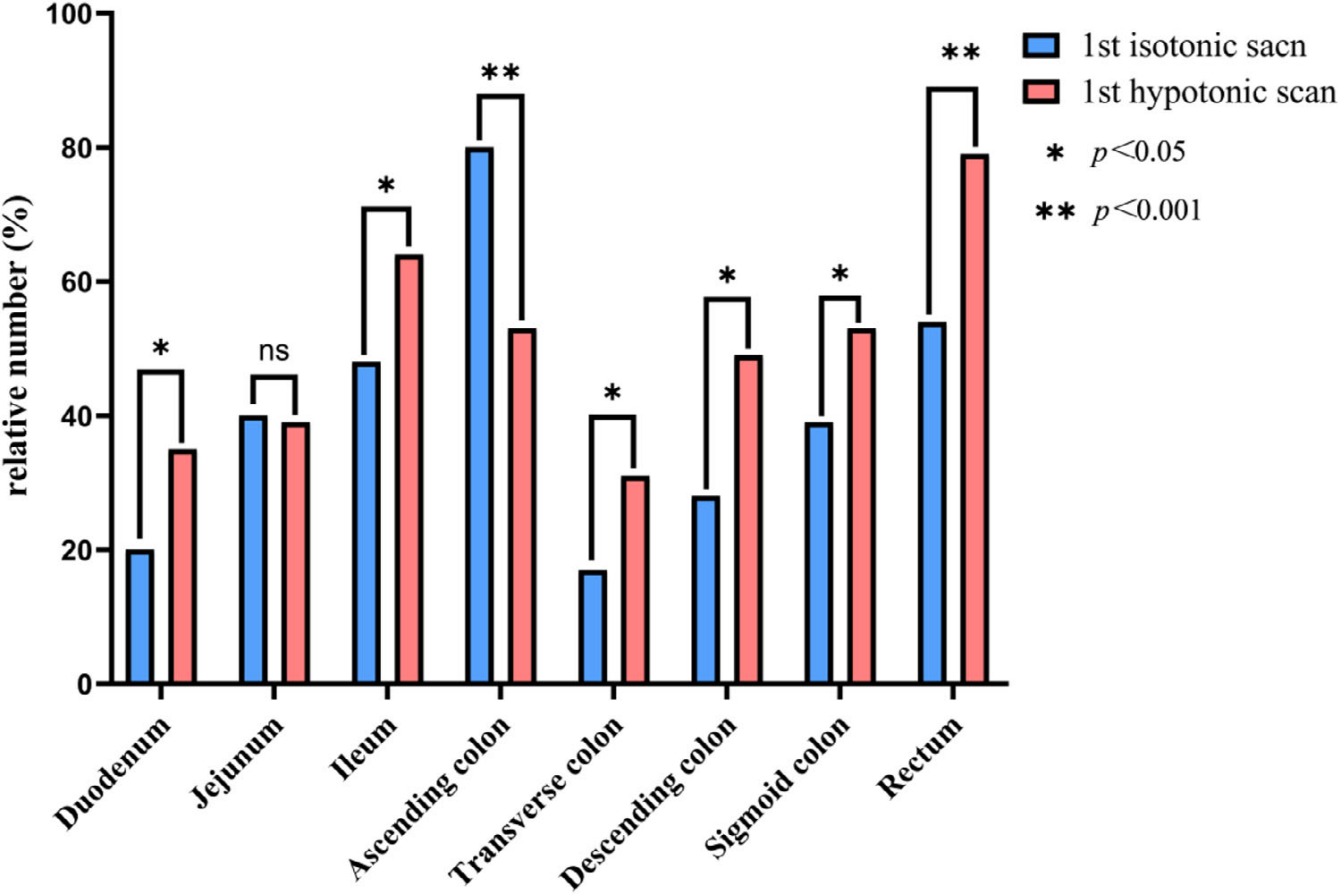
Incidence of physiological uptake in different intestinal segments between the first isotonic and the first hypotonic scans. NS: not significant. *p < 0.05, **p < 0.001.

No brown fat uptake was observed on PET/CT images in two groups. The [18F]‐FDG uptake in different bowel segments and TBR analysis in two groups are shown in Figure 1 and Table 2, respectively. In the isotonic group, the highest incidence of physiological uptake occurred in the ascending colon (80%), followed by the rectum (54%), ileum (49%), jejunum (48%), and transverse colon (17%). Meanwhile, in the hypotonic group, the highest prevalence of physiological uptake occurred in the rectum (79%), followed by the ileum (64%), ascending colon (53%), and sigmoid colon (53%), and transverse colon (31%). Compared with the hypotonic group, the isotonic group exhibited lower incidences of physiological uptake and reduced mean TBR in the duodenum, distal ileum, transverse colon, descending colon, sigmoid colon, and rectum. Although physiological uptake incidence in the jejunum showed no significant difference between two groups, the hypotonic group demonstrated decreased tendency of mean TBR in this segment. Notably, while the isotonic group showed higher physiological uptake incidence in the ascending colon, no significant difference was observed in the TBR in this segment between two groups.

**TABLE 2:** Target‐to‐background ratio of different intestinal segments in the first isotonic scan of isotonic group and the first hypotonic scan of hypotonic group.

	*N*	Duodenum	Jejunum	Ileum	Ascending colon	Transverse colon	Descending colon	Sigmoid colon	Rectum
Isotonic group	82	0.49 ± 0.13	0.56 ± 0.21	0.58 ± 0.23	0.87 ± 0.49	0.44 ± 0.29	0.50 ± 0.65	0.52 ± 0.28	0.58 ± 0.33
Hypotonic group	103	0.54 ± 0.14	0.63 ± 0.30	0.80 ± 0.36	0.75 ± 0.48	0.55 ± 0.26	0.65 ± 0.27	0.65 ± 0.27	0.78 ± 0.19
*p*‐value	–	0.012	0.066	<0.001	0.114	0.009	0.032	0.003	<0.001

Values are presented as mean ± standard deviation (SD).

### 3.3 Comparative analysis of [18F]‐ FDG uptake in intestine segments for patients receiving repeated examinations

**FIGURE 2:**
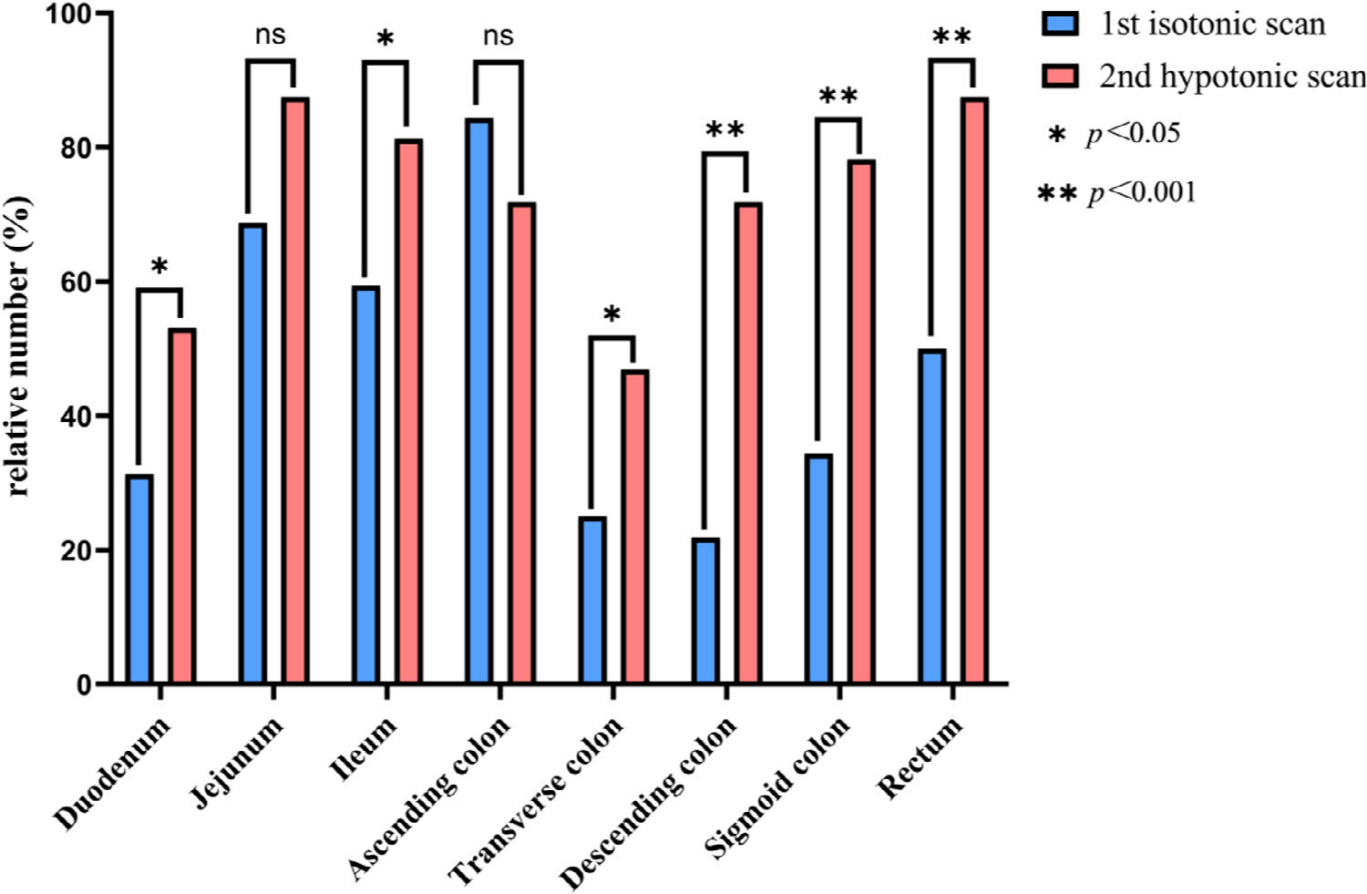
For patients with repeated PET/CT scans (first isotonic scan and the second hypotonic scan), incidence of physiological uptake in different intestinal segments. NS: not significant. *p < 0.05, **p < 0.001.

**FIGURE 3:**
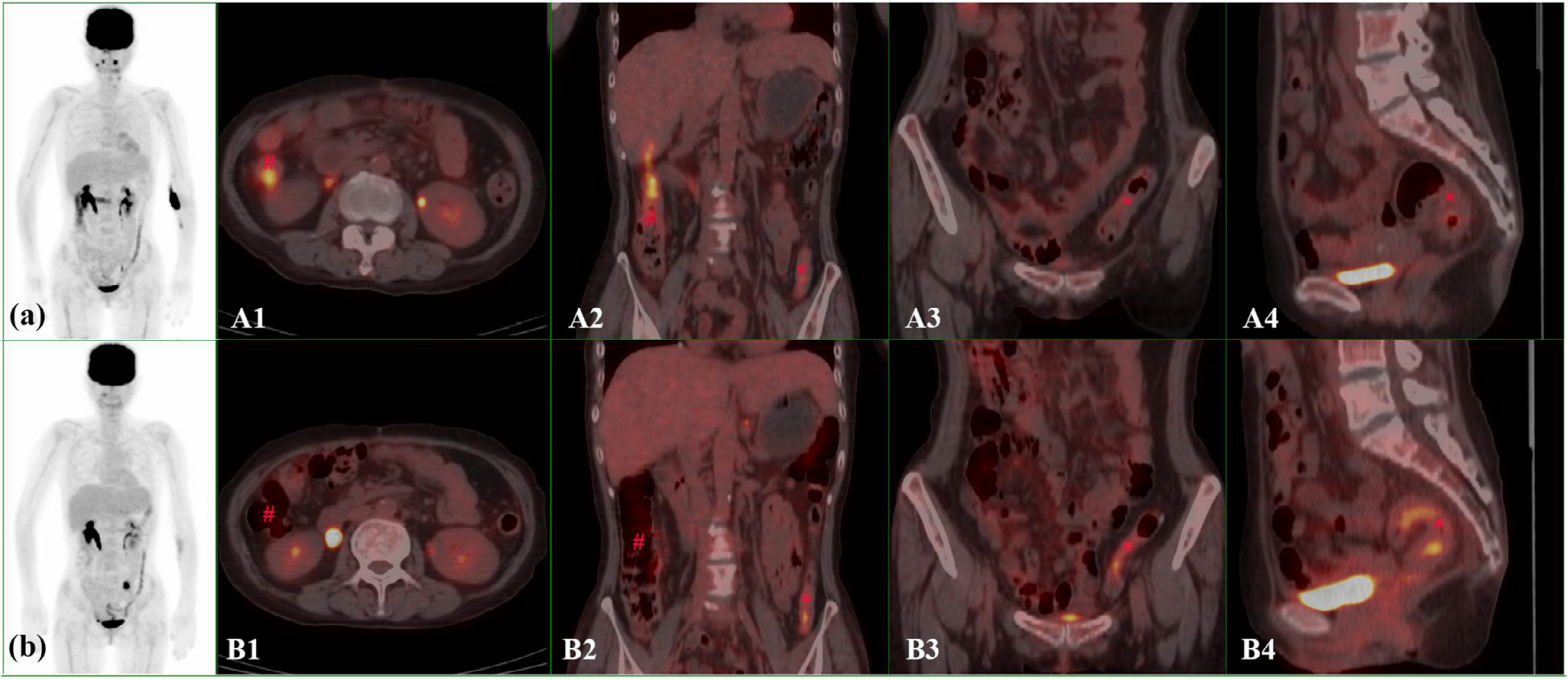
A shows the maximum intensity projection of a 37‐year‐old female patient with oral administration of isotonic saline before the examination, and B shows the maximum intensity projection of the same patient with oral administration of hypotonic water. # represented ascending colon. Compared with panel B, higher physiological uptake was observed in panel A. * represented descending colon, sigmoid colon to rectum, the physiological uptake in panel A was significantly lower than that in panel B.

In the isotonic group, 32 patients underwent repeated PET/CT examinations. The FDG uptake in different bowel segments and TBR analysis in first and second examinations are shown in Figure 2 and Table 3, respectively. The first examination (receiving isotonic saline) demonstrated reduced physiological uptake incidences in the duodenum (*p *< 0.05), jejunum (*p *< 0.05), ileum (*p *< 0.05), transverse colon (*p *< 0.05), descending colon (*p *< 0.001), sigmoid colon (*p *< 0.001), and rectum (*p *< 0.001) compared to the second examination. Meanwhile, mean TBR in in all these segments were also significantly decreased (*p *< 0.05). There were no significant differences in the physiological uptake incidence and the TBR in ascending colon between first and second examinations (*p *= 0.226, *p *= 0.664). One characteristic image is shown in Figure 3, the physiological uptake (except ascending colon) was obviously increased in the second examination (receiving hypotonic water), compared with the first examination (receiving isotonic saline).

**TABLE 3:** The intra‐individual differences of target‐to‐background ratio of different intestinal segments between the first isotonic scan and the second hypotonic scan in isotonic group.

	Drink before examination	Duodenum	Jejunum	Ileum	Ascending colon	Transverse colon	Descending colon	Sigmoid colon	Rectum
First	Isotonic saline	0.47 ± 0.09	0.60 ± 0.13	0.62 ± 0.01	0.89 ± 0.45	0.43 ± 0.01	0.37 ± 0.05	0.57 ± 0.01	0.94 ± 0.13
Second	Hypotonic water	0.52 ± 0.10	0.64 ± 0.01	0.88 ± 0.11	0.89 ± 0.51	0.53 ± 0.12	0.66 ± 0.05	0.65 ± 0.01	1.05 ± 0.33
*p*‐value	–	<0.001	<0.001	<0.001	>0.1	<0.001	<0.001	<0.001	0.005

Values are presented as mean ± standard deviation (SD).

In the hypotonic group, 19 patients underwent repeated PET/CT examinations. No significant differences were observed in physiological uptake incidences or mean TBR between the first hypotonic scan and the second hypotonic scan.

### 3.4 Factors associated with FDG uptake in intestine segments

As shown in Table 4, multiple linear regression analysis was performed to find possible factors associated with FDG uptake in the duodenum, proximal jejunum, distal ileum, transverse colon, descending colon, sigmoid colon, and rectum for 103 patients in the hypotonic group. BMI was negatively associated with FDG uptake intensity in the duodenum (*B* = −0.29, *p* < 0.05), jejunum (*B* = −0.32, *p* < 0.01), ileum (*B* = −0.31, *p* < 0.01), and sigmoid colon (*B* = −0.18, *p* < 0.05). Diarrhea was positively correlated with FDG uptake in the ascending colon (*B* = 0.24, *p* < 0.05), transverse colon (*B* = 0.21, *p* < 0.05), and rectum (*B* = 0.31, *p* < 0.01), whereas constipation showed a positive association with FDG uptake in the ileum (*B* = 0.21, *p* < 0.05). Bone tumors and gynecological tumors were positively correlated with FDG uptake in the ascending colon (*B* = 0.39, *p* < 0.05; *B* = 0.68, *p* < 0.05) and transverse colon (*B* = 0.17, *p* < 0.05; *B* = 0.24, *p* < 0.05). Additionally, blood glucose was negatively associated with FDG uptake in the duodenum (*B* = −0.19, *p* < 0.05). A statictically significant positive correlation was observed between gastroenteroscopy and FDG uptake in the transverse colon (*B* = 0.42, *p* < 0.01).

**TABLE 4:** Multiple linear regression analysis for the relevant factors of higher physiological intestinal uptake in hypotonic group.

	Duodenum	Jejunum	Ileum	Ascending colon	Transverse colon	Descending colon	Sigmoid colon	Rectum
Age beta (SE)	–	–	–	–	–	–	–	–
Sex beta (SE)	–	–	–	–	–	–	–	–
BMI beta (SE)	−0.29(0)^#^	−0.32(0.01)^##^	−0.31(0)^##^	–	–	–	−0.18(0.07)^#^	–
Diabetes beta (SE)	–	–	–	–	–	–	–	–
Glucose beta (SE)	−0.19(0.01)^#^	–	–	–	–	–	–	–
The FDG circulation time Beta (SE)	–	–	–	–	–	–	–	–
Constipation* beta (SE)	–	–	0.21(0.1)^#^	–	–	–	–	–
Diarrhea* beta (SE)	–	–	–	0.24(0.19)^#^	0.21(0.1)^#^	–	–	0.31(0.07)^##^
Bone tumors beta (SE)	–	–	–	0.39(0.15)^#^	0.17(0.08)^#^	–	–	–
Gynecological tumors beta (SE)	‐	–	–	0.68(0.23)^#^	0.24(0.12)^#^	–	–	–
Gastroenteroscopy* beta (SE)	–	–	–	–	0.42(0.12)^##^	–	–	–
Desmosis beta (SE)	–	–	0.55(0.19)^#^	–	–	–	–	–

Beta: regression coefficient, SE: standard error, only statistically significant items were showed, * within seven days prior to examination, ^#^ *p *< 0.05, ^##^ *p *< 0.001.

## 4 DISCUSSION

The physiologic ^18^F‐FDG uptake in intestines could disrupt precise interpretation of early intestinal tumor lesions and might mask true lesions, leading to either misdiagnosis or false‐positive outcomes.^21^, ^22^ Hence it is of great significance to regulate the uptake of ^18^F‐FDG in the intestines to reduce the risk of both false‐positive and false‐negative diagnoses.

The European Association of Nuclear Medicine (EANM) PET/CT tumor imaging guidelines indicated that water was associated with rapid reabsorption, and drinking water before the examination may lead to non‐specific FDG accumulation in the intestines. It is recommended to use low‐concentration positive contrast agents.^23^ To verify this finding, we conducted this study. To our knowledge, this is the first investigation comparing physiological ^18^F‐FDG uptake incidence in different intestinal segments between patients who received isotonic saline and those who received hypotonic water. In this study, we first compared the SUVmax and TBR of various intestinal segments between patients receiving isotonic saline and hypotonic water. Secondly, we performed a comparative study on patients undergoing repeated PET examinations to validate our results. Our findings demonstrated that pre‐examination administration of isotonic saline reduced physiological ^18^F‐FDG uptake in most intestinal segments (duodenum, distal ileum, transverse colon, descending colon, sigmoid colon, and rectum), and one characteristic imaging was shown in Figure 3. These differences may be attributed to differences in osmotic pressure between these two oral agents. A 2015 study revealed that oral isotonic mannitol decreased physiological ^18^F‐FDG uptake compared with hypotonic water, enhancing gastrointestinal dilation and ultimately improving image quality.^13^ Compared with hypotonic water, isotonic saline showed reduced absorption in the intestines, promoting intestinal dilation and diminishing physiological ^18^F‐FDG uptake. However, we also identified unexpectedly high uptake in the ascending colon in patients receiving isotonic saline. This unique discrepancy in the ascending colon might stem from varying absorption rates of isotonic saline across different intestinal segments,^24^ warranting further investigation into the underlying mechanism. Consequently, isotonic saline was regarded as an effective negative contrast agent for intestinal segments except ascending colon, and its influence still remained questionable for ascending colon.

We further explored the potential factors associated with physiologic intestine uptake of ^18^F‐FDG in patients receiving hypotonic water. Multiple regression analysis showed that constipation had a positive effect on FDG uptake in distal ileum, and diarrhea had a positive effect on FDG uptake in transverse colon, ascending colon, and rectum. Our results were consistent with previous studies.^25^ The abnormal intestinal activity (diarrhea and constipation) was associated with glucose metabolism, which was probably due to the abnormal smooth muscle movements.^26^ We also noticed that the BMI has a negative effect on FDG effect by duodenum, distal ileum, proximal jejunum, and sigmoid colon, which indicated that for patients with low BMI, nuclear medicine physicians should pay more attention to differentiate malignant FDG uptake in these intestinal sections. However, the underlying mechanism required further investigation. One study reported lower BMI patients has an increased FDG uptake in the neck, shoulder region.^27^ It was reported that low BMI patients are more prone to feel cold, which led to stimulation of the sympathetic nerve and further increased glucose uptake.^28^ Moreover, another study reported that intestinal glucose uptake was increased in patients with a history of upper gastrointestinal and bariatric surgery.^20^ It should be noted that the active transport of tracers into the intestine through sodium‐glucose cotransporters, intestinal flora, intestinal wall inflammatory cells and other factors might also lead to changes in intestinal uptake.^29^, ^30^ However, conducting parallel and longitudinal analyses of PET‐CT‐derived glucose uptake measurements in intestinal segments to elucidate these relationships remained challenging in human subjects. We also noticed that bone tumors and gynecological tumors were positively correlated with FDG uptake in the ascending colon and transverse colon, whereas the underlying mechanism required further investigation.

Some limitations existed in this study. Firstly, the underlying mechanisms by which drinking isotonic saline was associated with intestinal FDG uptake still remains unclear. Moreover, due to the necessity of ensuring adequate hydration prior to the examination, the control group that received no intervention was not established. Consequently, it remains unclear whether isotonic saline provides superior improvement in background suppression compared to no preparation at all. Finally, more patients from a larger region are still needed to verify our conclusions.

## 5 CONCLUSION

Oral isotonic saline resulted in lower physiologic ^18^F‐FDG uptake in different intestine segments (except ascending colon), which may pave the way to more accurate assessment of gastrointestinal diseases.

## AUTHOR CONTRIBUTIONS

*Conceptualization*: Qian Wang and Yuan Li. *Formal analysis*: Shijia Weng and Xiaoren Liu. *Methodology*: Shijia Weng and Yuan Li. *Data curation*: Shijia Weng and Xiaoren Liu. *Writing—original draft*: Shijia Weng. *Writing—review and editing*: Qian Wang and Yuan Li. All authors contributed to the article and approved the submitted version.

## CONFLICT OF INTEREST STATEMENT

The authors declare that they have no known competing financial interests or personal relationships that could have appeared to influence the work reported in this paper.

## ETHICS STATEMENT

The study was carried out in accordance with the guidelines of the Declaration of Helsinki. This study was approved by the Ethics Committee of Peking University People's Hospital. All participating patients have duly signed informed consent forms.

## Data Availability

The datasets used and analyzed during the current study are available from the corresponding authors on reasonable request.
